# The Athlete’s Perception of Coaches’ Behavior Towards Competitors with a Different Sports Level

**DOI:** 10.2478/hukin-2013-0086

**Published:** 2013-12-31

**Authors:** Małgorzata Siekanska, Jan Blecharz, Agnieszka Wojtowicz

**Affiliations:** 1 Department of Psychology, University School of Physical Education in Cracow, Poland.

**Keywords:** the Pygmalion effect in sport, self-fulfilling prophecy, perception of coaches’ behaviors

## Abstract

The study was designed to examine how active and former athletes across a different sports level perceived coaching behavior. Eighty competitive athletes (44 males and 36 females; 21.89 ± 1.48 years of age; 8.35 ± 3.65 years of competitive experience) from the University School of Physical Education in Cracow, Poland, participated in the study. They represented both individual (n = 50) and team sports (n = 30). Seventeen participants were internationally renowned and 63 were recognized for competitive excellence at a national level. The participants responded to a demographic survey and the Coaches’ Behaviors Survey. The qualitative analysis procedures were employed to extract themes from open-ended questions. It was confirmed that coaches who perceived their athletes as more skilled, also treated them differently. Female athletes as compared with male athletes, more frequently pointed at the leniency in coach’s behavior towards highly skilled athletes, and perceived it as a factor inhibiting athletic development. Additionally, women often found individualization of the training process as a behavior reinforcing development. Less accomplished athletes more often pointed out to “a post-training session interest in the athlete” as directed only towards more accomplished counterparts; however, they indicated “leniency and favoring” less often than the athletes with international achievements. They also listed “excessive criticism” as a type of behavior hindering development, but they indicated coaches’ “authoritarianism and distance” less frequently than the more accomplished counterparts. The study added data to the discussion of the Pygmalion effect and the phenomenon of the self-fulfilling prophecy both in general (Rosenthal and Jacobson, 1968; Harris and Rosenthal, 1985; Jussim, 1989) and sport psychology (Harris and Rosenthal, 1985; Horn et al., 1998; Solomon and Kosmitzki, 1996; Solomon et al., 1998; Solomon, 2001).

## Introduction

The important role of a coach in competitive sports is self-evident. A good coach is responsible for supporting physical, mental, technical and tactical development of athletes, so that they can achieve their highest goals (Becker, 2009). However, the answer to the question what makes the athlete-coach interaction well-balanced does not seem so obvious. Although much research has already been carried out into this area, many issues still remain unexplained. It may be caused mainly by the fact that researchers explore only “measureable” forms of coaching behaviors (observable, replicable, etc.), and seldom provide insight into the experiences of the athlete connected with being coached (Becker, 2009). Therefore, this study was designed to describe athletes’ perceptions of coaches’ behavior towards athletes of a different sports level.

### The Pygmalion effect

Similarity between the coach–athlete and teacher-student relationships, especially the way of communicating and its influence on the athlete’s motivation and performance (known as the Pygmalion effect [e.g., Merton, 1948]), has been well documented (Chelladurai et al., 1999; Martin et al., 2009; Turman, 2001, 2003). Therefore, it seemed relevant to explore the effects found in the academic contexts in the domain of sport.

Rosenthal and Jacobson (1968) referred to this phenomenon as the teacher’s, or researcher’s or practitioner’s expectancy effect. In their study, they carried out intelligence tests on primary school students and identified a group of subjects who scored best on tests, and finally informed teachers about the targeted high-achievers in their classes. In fact, the students were selected at random and their scores were not taken into consideration in labeling the high-achiever group. At the end of the school year it was found out that many of the targeted subjects (as high-achievers) improved their grades and did better on intelligence tests than their peers. In other words, if a teacher believed that a student was gifted, their behavior changed (e.g., the teacher was more attentive to students, provided them with extra practice and motivated more than others). In turn, the students tried to conform to the teacher’s expectations, and as a result showed bigger gains in their academic achievements. Indeed, there were factors in interpersonal contact, which from the very beginning influenced the formation of expectations concerning the participants and the interaction itself. They comprised external factors (e.g., appearance, information about participants obtained earlier) and internal factors (e.g., traits of temperament, experiences, and attitudes of participants). As time went by, the expectations became modified and reinforced. As a result, the teachers developed a relationship/belief, which influenced their behavior in order to meet the initial expectations: (a) the Galatea effect (based on positive expectations), and (b) Golem’s effect (based on negative expectations; Babad et al., 1982).

Skarzynska (1981) explored teachers’ behavior towards gifted and non-gifted students at school and whether the teacher’s opinions about their students’ competence would affect the teacher’s grading and type of help provided and instruction. One group of participants was informed they would teach gifted, and the other group non-gifted students. In fact, ‘gifted’ and ‘non-gifted’ students were the study collaborators (i.e., researcher’s assistants) and they all behaved in the same way (e.g., made the same number of similar mistakes). The results of the study were congruent with Rosenthal and Jacobson’s (1968) findings. For example, the students labeled as gifted, at the moment of making a mistake, were provided with feedback information containing a proper solution to a problem, whereas those labeled as non-gifted received messages referring to motivation. Also, when the high-achievers proposed correct solutions to a problem, they received positive grades more often than the remaining students. The study confirmed findings that students who were perceived as gifted, more frequently than the non-gifted, heard comments on their grades and received additional feedback on their progress in learning. Furthermore, the study revealed that the gifted participants were perceived as more friendly than the underachievers, and the level of their performance was often overestimated as compared to the underachievers whose performance was often underestimated. The researcher concluded that it was an example of the self-fulfilling prophecy. In other words, the structure of the teacher’s behavior was modified by their perception of the student’s competence, and therefore, the teacher’s instruction differed depending on the type of student they perceived that they dealt with. As a consequence of these situational differences (not competence differences) a student labeled as non-gifted made slower learning progress than the one perceived as a gifted one (Skarzynska, 1981), which was confirmed in many other studies (Martinek, 1988, 1989; Jussim, 1989).

### The Pygmalion effect in sport

Studies on the self-fulfilling prophecy and the Pygmalion effect inspired other researchers, including those in competitive sports. Coaches, similarly to teachers, form expectations about their athletes based on their physical appearance, personality or information concerning their past achievements, behavior during training sessions or scores on skill tests (Short and Short, 2005). Expectations can also be based on stereotypes regarding body build, height, race or socioeconomic status (Horn et al., 1998). The coach’s expectations affect the quality of the coach-athlete interactions, kind of instructions and type and frequency of feedback. As a result, low-expectancy players receive less support from their coaches and have fewer opportunities to show their real skills. Solomon et al. (1998) conducted research on possible differences in communication between coaches and low as well as high-expectancy players. The study revealed that at the beginning of a competitive season low-expectancy players received more feedback; however, as time passed by, more information on technical aspects of the game was given to the high-expectancy players. The difference became more apparent at the end of the season, thus indicating that coaches adjusted and modified their ways of communicating with athletes, especially in key moments during the season.

In another study, Solomon (2001) investigated whether coaches, in relation to their expectations, were able to anticipate their athletes’ achievements. Her findings showed that in this case only the coach’s evaluation of the athlete’s self-confidence was a reliable predictor of a sports achievement, other expectations turned out unrelated. It might be speculated that coaches too often overestimated their own abilities to assess athlete’s performance, which in turn could produce wrong patterns of the coach-athlete interactions. If the coach evaluated the athlete’s skills inaccurately, then even gifted players might never show their real potential (Short and Short, 2005). Furthermore, researches indicated that the coach’s opinion about the athlete’s skill levels remained unchanged over the course of the season and unaffected by their athletes’ performances (Solomon and Kosmitzki, 1996; Solomon, 1998). Another study (Trouilloud et al., 2002) revealed that PE teachers formed expectations at the beginning of the school year and determined their final opinions about their students, their self-efficacy and athletic abilities and skills.

The outcomes of a variety of studies conducted among students, athletes and coaches enabled researchers to propose a sequence of four steps in the self-fulfilling prophecy (Martinek, 1981; Brophy, 1983; Harris and Rosenthal, 1985; Jussim, 1986). At first, the coach formed expectations of each athlete and determined the level of performance that they would reach over the course of a season. Secondly, the coach’s expectations affected their behavior and their treatment of the athlete in accordance with their sports level. In the third step of the sequence, the coach’s behavior influenced the athlete’s performance and the speed of learning, which in turn affected the athlete’s self-esteem, motivation and performance expectations, and, ultimately, their achievements. Finally, the athlete’s behavior and performance conformed to the coach’s expectations and they reinforced the coach’s belief that their initial judgments were accurate, and the process continued.

### Objectives

The studies on the self-fulfilling prophecy and the Pygmalion effect usually concentrated on coaches, their expectations towards athletes and the coach-athlete interactions depending on the athlete’s achievements, but they seldom showed the situation from the athlete’s point of view.

The primary objective of the study focused on determining whether the athlete perceived any differences in coaching behaviors depending on whether the coach worked with a high or low-expectancy athlete, and what kind of behaviors they were.

The second objective concerned acquiring an answer to the question of what behaviors did athletes perceive as enhancing or inhibiting their sports development.

The third objective dealt with the relationships between the types of behavior in the coach-athlete interactions and gender, the athlete’s achievements, the type of sport, the stage of the athlete’s competitive career and the number of the coaches they had worked with, as well as the athlete’s age.

## Material and Methods

### Participants

The study included 80 athletes (48 active and 32 former) of the Faculty of Physical Education and Sport at the University School of Physical Education in Cracow, Poland (44 males and 36 females), who represented different, both individual (50 persons), and team (30 persons) sports. Athletes aged 21.89 ± 1.48 took part in the study and their average training experience equaled 8.35 ± 3.65 years. Based on their sports achievements, the participants were divided into international (N=17) and national sports level (N=63). The internationally renowned athletes were included in the high-performance group while the nationally recognized ones in the low-performance group.

### Measures

All the participants were asked to answer questions in the Coaches’ Behaviors Survey which allowed for generating factors in the coach-athlete interaction and showing differences in the treatment of high-achievers (Appendix No 1). The survey consisted of two parts. The first part dealt with questions about personal details and sports achievements in which the participants were also asked to state to what degree the coach-athlete interaction affected their achievements and sports development (0–100%).

Part two contained exploratory questions. The first one addressed the participants’ opinions about the coach’s behavior towards athletes with different sports skills. In case of giving a positive answer, the participants were asked to substantiate their opinions, that is to describe manifestations of behavior which proved that the coach favored those athletes whom they considered to be more talented. The last two were open-ended questions and concerned the coach’s behaviors towards athletes. The participants were asked to enumerate behaviors in favor of the athlete’s development and behaviors which hindered it. The respondents could mention any number of behaviors.

### Procedure

The survey was anonymous and was carried out in four stages (sets). The participants wrote down their answers on appropriate forms.

### Data Analysis

The statistical analysis was conducted employing the *Statistica 8.0* software. Basic descriptive statistical data were calculated for the analyzed quantitative variables and the percentage values were calculated for the qualitative variables. Mainly non-parametric statistics (i.e. the chi^2^ test) were employed for the assessment of the relation between two nominal variables and the U Mann-Whitney test, in case of lack of normal distribution, checked by the Shapiro-Wilk test. In case of the remaining comparisons the analysis of variance for interactions and the *t*-test were used. The results where *p* was smaller than the accepted level of significance α=0.05 were considered statistically significant. The qualitative analysis in the form of exploratory categorizing responses to open-ended questions in the Coaches’ Behaviors Survey, was also employed.

## Results

The results were divided into four main parts. The first part contained results of the qualitative analysis from the Coaches’ Behaviors Survey. The second part dealt with gender differences with regard to the number and type of the coach-athlete behaviors. The third part concerned the analysis of the relationship between dependent variables and the sports level. The last part contained analyses which were interesting due to exploratory reasons and had not been described beforehand.

### Analysis of qualitative data

To analyze the answers to the open-ended questions in the survey, in stage one the analysts were paying special attention to descriptions pointing out to specific behaviors which could be observed (e.g. *the coach informed the athlete about mistakes without showing how to correct or improve the execution of a given skill*), and excluded those pieces of information which dealt with the general assessment of behavior (e.g. *the coach delivered a bad training session)* and interpretation (e.g. *the coach was not prepared to deliver a training session)*. Stage two consisted of exploratory categorizing the content of the answers from respondents (Stemplewska-Zakowicz and Krejtz, 2009; Gibbs, 2007). Two competent judges independently revised the survey material paying special attention to: 1) the observed similarities, and 2) possible distinct (unique) categories. Labels were attached to the pre-selected and emerged categories. Once the consensus on the categories between the two analysts was reached, they were used for subsequent coding. After one month this procedure was repeated to verify the categories created earlier, thus, enhancing reliability in the data coding.

As presented in Table 1, most frequently, the participants indicated that a coach paid more attention and devoted more time to high-achieving athletes. The least frequent category attributed to the coach behaviors towards high-achievers was a good coach-athlete interaction, mentioned only by seven participants.

The most often reported behaviors enhancing sports development were good coach-athlete interactions and individualization of a training process (Table 2). The least frequent behaviors, pointed out only by twelve participants, were the coach’s control and error correction.

Finally, the most frequently selected behaviors inhibiting athletic progress were poor coach-athlete interactions and lack of coaching competence (Table 3). The least frequent behavior, mentioned only by three participants, was the coach’s leniency.

### Gender differences

The analyses revealed that males, more often than females, pointed out to “leniency and favoring” as the behavior of a coach towards athletes with better sports performance (chi^2^=5.518; df=1; p=0.019). Also, male athletes selected “control and error correction” more frequently as behaviors enhancing athletic development (chi^2^=6.526; df=1; p=0.011), but less often than females chose “individualization of training sessions” (chi^2^=6.485; df=1; p=0.011). On the other hand, females pointed out to “excessive leniency” more often than males (chi^2^ NW=4.934; df=1; p=0.026) and “lack of good relation and spirit” as behaviors inhibiting athletic development (chi^2^=4.246; df=1; p=0.039). Gender differences in response to the remaining items were not statistically significant.

### Analyses connected with the participants’ sports level

Other analyses concerned discrepancies between individuals with different levels of sports achievements and the frequency of selecting particular categories of behaviors in the coach-athlete interactions. The athletes characterized by the lowest sports performance more often pointed to “a post-training session interest in the athlete” as the behavior of a coach directed only to high achievers (chi^2^=4.982; df=1; p=0.026). However, they mentioned “leniency and favoring” (chi^2^=5.711; df=1; p=0.017) less often than renowned athletes. In relation to the behaviors enhancing or inhibiting athletic progress they selected “excessive criticism” as the behavior inhibiting their progress (chi^2^=7.684; df=1; p=0.006) more frequently than high-achieving counterparts. However, they pointed out to authoritarianism, formalism, indifference and distance less often than the athletes with international achievements (chi^2^=4.901; df=1; p=0.027). Other differences were not statistically significant.

### Other exploratory analyses

**Figure 1:**
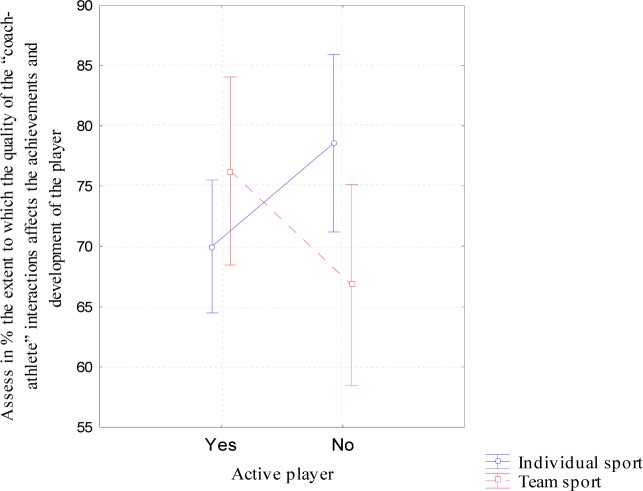
Interaction of type of sport and phase of sports career

Other analyses concerned relationships between the coach’s behaviors identified in the qualitative analysis and (a) the assessment of the degree of influence of the coach-athlete interactions on the type of sport practiced, (b) the phase of the competitor’s career and (c) the number of coaches the athlete had worked with and the age of participants. It was found that the assessed difference in the coach-athlete interactions, which affected the participants’ performance and development in both the team and individual sports, was modified by the phase of their sports career (F=5.993; df=1.76; p=0.017; Figure 1). No statistically significant differences were found in the assessment of the influence of the coach-athlete interactions on the development of active athletes (F=1.698; df=1.76; p=0.197). However, active and former individual sport athletes scored higher in influence of that interaction on sports development than team sport participants (F=1.445; df=1.76; p=0.038).

Also analyzed were the differences in the number of indicated categories of the coach-athlete behaviors between individual and team sports participants, and whether they were modified by the phase of their sports career. No statistically significant differences were found in the number of indicated categories of behaviors demonstrating different attitude towards the athletes with higher achievements (F=0.990; df=1.76; p=0.323), the number of categories of behaviors enhancing the athlete’s sports development (F=0.923; df=1.76; p=0.340) and the categories of behaviors inhibiting athletic progress (F=3.043; df=1.76; p=0.085).

Finally, all the categories of coaching behaviors in the coach-athlete interactions were analyzed for exploratory purposes. The first analysis took into consideration the link between the sports experience and the frequency of indicating particular categories of behaviors in the coach-athlete interactions towards high-expectancy athletes. The participants characterized by a longer sports experience more often pointed out to “leniency and favoring” (t=2.299; df=78; p=0.024). Other explored differences were not statistically significant.

Subsequent analyses concerned the relationship between the length of sports experience and the frequency of indicating particular categories of behaviors in the coach-athlete interactions as enhancing and inhibiting the athletes’ progress. No statistically significant differences were discovered.

The frequency of selecting particular categories of behaviors in the coach-athlete interactions and the participants’ different phases of their sports careers were also compared. The data revealed that active athletes, more often than former athletes, perceived “leniency and favoring” as a coaching behavior directed only to the athletes with higher achievements (chi^2^=11.524; df=1; p=0.001). In relation to the behaviors enhancing or inhibiting athletic progress, active athletes selected “control and error correction” more often as enhancing the athlete’s progress, and indicated individualization of training sessions less often than the former athletes (chi^2^=4.827; df=1; p=0.028). In case of behaviors inhibiting athletic progress, active athletes referred to excessive informality in the coach-athlete interactions (chi^2^=5.114; df=1; p=0.024). Other differences were not statistically significant.

Subsequently, the relationship between age and the frequency of choosing particular categories of behaviors in the coach-athlete interactions was analyzed. Younger athletes pointed out to “leniency and favoring” as the coaching behavior directed only to high achievers less often than the older ones (U=515.500; Z=−2.674; p=0.007). However, they perceived “personalization of training sessions” as the coaching behavior promoting the athlete’s progress more often than their older counterparts (U=554.500; Z=2.326; p=0.020). Other differences were statistically not significant.

Another analysis referred to a relationship between the frequency of selecting particular categories of behaviors in the coach-athlete interactions and the number of coaches the participants had worked with. The data revealed that the participants who had worked with a greater number of coaches, indicated “leniency and favoring” as a sign of different treatment of more skilled athletes (U=539.500; Z=2.442; p=0.015). Other relationships did not reach a statistically significant threshold.

An additional analysis was performed taking into account the relationship between the type of sport (individual vs. team) and the number of coaches the participants had worked with. It was found that team sport athletes worked with a greater number of coaches than individual sport athletes (U=398.500; Z=−3.493; p<0.001).

## Discussion

The objective of this research was to provide a description of the Pygmalion effect from the athlete’s point of view. Ninety per cent of participants responded positively to the question: “Do you think the coach’s attitude towards the athlete with higher sports level differs from the one directed towards low-achievers?” This assertion meant that irrespective of the type of sport, level of achievements, and the length of sports experience athletes recognized a different coaching behavior towards competitors whom coaches considered as highly skilled. The result was congruent with the four-step sequence of the Pygmalion effect (Martinek, 1981). Interestingly, the outcomes concerning gender differences in perception of the coach-athlete interactions revealed that females were ready to build up a relationship and spend time with other members of their team more often than males. Therefore, they paid more attention to coaching behaviors such as maintaining good spirit and personalizing training sessions. On the other hand, males focused more on such factors as control and error correction because they served as specific clues to peak performance. Furthermore, the research revealed that there were higher levels of extrinsic motivation in male than in female athletes (Monazami et al., 2012). However, behaviors limited strictly to the emotional side of the coach-athlete interactions were appreciated to a greater extent by females than by males. This also confirmed gender differences in the training process described by Miller et al. (2008), where males put more value on feedback and technical instructions, whereas females, exhibited more need for emotion-directed actions and held a strong belief in a coach.

Females, less often than males, identified leniency as the coach’s behavior directed to high performance athletes, but defined it as the one hindering athletic development. It meant that in the perception of female athletes the Pygmalion effect did not always produce desirable effects. According to the notion of the sequence of four steps of the self-fulfilling prophecy, in a coaching behavior congruent with their expectations for an athlete, the final response of the athlete should finally conform to the coach’s expectations (Martinek, 1981; Brophy, 1983; Harris and Rosenthal, 1985; Jussim, 1986). However, in this case coaching behavior towards more skilled athletes did not result in their enhanced sports growth, but actually in its perceived hindrance. Furthermore, the coach’s leniency and favoring more talented players were perceived by the top level achievers in sports. We may only speculate whether they indeed perceived or experienced such behaviors. Moreover, active athletes noticed leniency and favoring more often than the former ones. The former athletes also pointed out to the fact that such coaching behaviors did not promote athletic progress.

Males, more often than females, indicated control and error correction as the coach’s behavior favoring more talented athletes. At the same time, individualization of training sessions was for male athletes the factor, which improved their athletic development. Furthermore, contrary to Konter’s results (2007), it was found that male athletes paid more attention to expert’s competence than female athletes.

The level of sports achievements also differentiated the perception of what hindered athletic development. Athletes with lower sports levels pointed out to excessive criticism, but not their renowned counterparts. This difference can be related to a different interpretation of coach’s behavior. Many factors could affect the athlete’s understanding of the coach’s behaviors. It could result, for example, from the athletes’ self-assessment, which in turn influenced their interpretation of messages, which the coach sent about him or her as a person. If self-assessment was low, even an error correction message could be interpreted by an athlete as an attack on their ego, and could automatically activate the defense mechanisms. In that case, even feedback, which was meant to be constructive, might be rejected and interpreted as groundless criticism. In Kenow and Williams’ (1992) and Konter’s (2009) studies on the coach’s perception by anxious and lacking in self-confidence female players, the participants assessed negatively most of the coach’s behaviors. Competitors from the less-accomplished youth league more often concentrated on negative and critical comments of their coach, and different punishments (e.g. giving less playing time).

Interestingly, active athletes (both individual and team sports), similarly appraised the influence of the coach-athlete interactions on their sports progress and achievements. However, this perception changed when the athletes finished their sports career. The former individual-sport-athletes scored higher when assessing the effect of the coach-athlete interactions on their sport results than the former team-sport-athletes did. It might be related to a greater self-awareness among individual sport athletes developed with time. The athletes of team sports, however, concentrated more on team functioning and cooperating with other members of the team – the issues their coaches paid a special attention to (Solomon and Rhea, 2008). Conversely, in individual sports it was the self-focus that seemed to be of primary importance. Furthermore, the relationship with a coach differed, and it affected the way a former athlete perceived it afterwards. Specifically, former athletes attached greater importance to it, as in their view the success or failure depended entirely on the athlete and their coach. Lorimer and Jowett (2009) stated that a higher concurrence exists between the coach’s and the athlete’s feelings during a training session in individual sports. In other words, the level of empathy between the two was higher than in the coach-athlete interaction in team sports. The individuals who used to be involved in team sports, when they looked back at their sports career, more often directed their attention to their team and its members, than to their personal involvement. They also experienced more frequent changes of a coach in their careers, and therefore, the coach-player interaction was considered less important to them The positive outcomes related to the change of a coach and discontinuing contact with the previous coach can be observed for example in soccer - as the performance of the team temporarily improves after a new coach is assigned (Lago-Peñas, 2011). The relationship described here did not occur between individual and team sport athletes, and therefore, it was speculated that those differences became observable for athletes retrospectively, that is only after they had managed to look back and analyze their careers more thoroughly.

## Conclusions and practical implications

The results of the present study identified problematic areas in the current understanding of the Pygmalion effect, which require future analysis. One of the unique findings suggests that the high-expectancy athletes may perceive the coaching behavior as inhibiting (rather than enhancing) their athletic progress. It is commonly known that false assumptions on the athlete’s performance potential may bring negative effects on the actual performance outcomes. It could mainly concern exerting too great pressure and demands on athletes. The behavior from the category of leniency and favoring, which works on the assumption of reducing pressure and facilitating development, has been assessed by the competitors as a developmental inhibitor. Clearly, research on the coach-athlete interactions from the perspective of an athlete needs to be continued. The measures designed to assess behaviors in the coach-athlete interactions used in the present study might become a useful tool in future research.

Additionally, we can conclude that there are a lot of factors indirectly related to the athlete’s skills and abilities which significantly affect their sports achievements. Thus, in order to promote the broad development of the athlete, we should adopt an interdisciplinary approach, which takes all enhancing or hindering aspects of athletic development into consideration (Zdebski and Blecharz, 2004). While training a group of athletes one should act in such a way that each and every competitor would feel that he or she is a member of a team and an important component (Hodge, 2004). Regardless of team or individual sports, it is vital to plan a training session in such a way that it includes tasks for both more and less talented athletes (Ericsson, 2003). The time and attention devoted to training sessions – as opposed to information or money – are limited reserves and that is why the coach should manage them well (Davenport and Beck, 2001). However, it is exactly these reserves, which are differently used by the coach towards more and less talented athletes. Apparently, the less talented (or low-expectancy) athletes scarcely benefit from these resources. In such a case, the athlete should find a way to draw the coach’s attention for example, by asking for more instructions or additional feedback on their performance during a training session. Such behaviors can increase a positive image of the athlete, and consequently reduce the negative impact of the Pygmalion effect (Aronson, 1999).

## Figures and Tables

**Table 1:** Categories of manifestations of different approaches to more talented athletes

Categories of manifestations of different approaches to more talented athletes	No.	%
1. Devotes more time	41	68.33
2. Other types of training tasks	9	15.00
3. Post-training session interest in the athlete	14	23.33
4. Leniency and favoring	24	40.00
5. Higher expectations / requirements	9	15.00
6. Good coach-athlete interactions	7	11.67

**Table 2:** Behaviors in the coach-athlete interactions enhancing a complete athletic development

Behaviors in the coach-athlete interactions enhancing a complete athletic development	No.	%
1. Control and error correction	12	20.00
2. Personalization of training processes	37	62.00
3. Partner-like behaviors, but discipline maintained	25	41.67
4. Coach’s professionalism	28	47.00
5. Interest in the athlete and his or her private life	29	48.00
6. Good coach-athlete interactions	38	63.33

**Table 3:** Behaviors in the coach-athlete interactions inhibiting athletic development

Behaviors in the coach-athlete interactions inhibiting athletic development	N	%
1. Exerting pressure	13	21.67
2.Excessive favoring in a team	10	17.00
3 Big informality in the coach-athlete interactions	5	8.33
4.Excessive criticism	20	33.33
5.Excessive leniency	3	5.00
6. Behaviors revealing lack of coach’s professional competence	31	51.67
7. Authoritarianism and overbearing	21	35.00
8. Lack of good spirit and interaction	39	65.00

## Appendix no. 1 Coaches’ Behaviors Survey

### Part I

Sex: F/M

Age……

Sports discipline…………

Athletic competition experience ………….

How many coaches have you worked with so far? …………….

Are you still an active competitor? YES NO

Assess in % to what degree the coach-athlete interaction affects the athlete’s achievements and development (0% min; 100% max.)………%

### Part II

1. Do you think the coach’s attitude towards the athlete believed to have higher achievements differs from the one directed to a less talented athlete? (YES/NO) If so, how does it manifest?
2. List coaching behaviors which promote a complete development of an athlete.

List behaviors in the coach-athlete interactions which inhibit a complete development of an athlete.
